# Roles of the Na^+^/H^+^ Exchanger Isoform 1 and Urokinase in Prostate Cancer Cell Migration and Invasion

**DOI:** 10.3390/ijms222413263

**Published:** 2021-12-09

**Authors:** Xiuju Li, Benjamin Buckley, Konstantin Stoletov, Yang Jing, Marie Ranson, John D. Lewis, Mike Kelso, Larry Fliegel

**Affiliations:** 1 Department of Biochemistry, University of Alberta, Edmonton, AB T6G 2H7, Canada; xjli@ualberta.ca (X.L.); yangjinghist@163.com (Y.J.); 2 Illawarra Health and Medical Research Institute, Wollongong, NSW 2522, Australia; bbuckley@uow.edu.au (B.B.); mranson@uow.edu.au (M.R.); mkelso@uow.edu.au (M.K.); 3 School of Chemistry and Molecular Bioscience, University of Wollongong, Wollongong, NSW 2522, Australia; 4 Molecular Horizons, University of Wollongong, Wollongong, NSW 2522, Australia; 5 Department of Oncology, University of Alberta, Edmonton, AB T6G 2H7, Canada; kstoletov@gmail.com (K.S.); jdlewis@ualberta.ca (J.D.L.)

**Keywords:** acid extrusion, amiloride, metastasis, Na^+^/H^+^ exchanger, prostate cancer, proton transport, urokinase plasminogen activator

## Abstract

Prostate cancer is a leading cause of cancer-associated deaths in men over 60 years of age. Most patients are killed by tumor metastasis. Recent evidence has implicated a role of the tumor microenvironment and urokinase plasminogen activator (uPA) in cancer cell migration, invasion, and metastasis. Here, we examine the role of the Na^+^/H^+^ exchanger isoform 1 (NHE1) and uPA in DU 145 prostate cancer cell migration and colony formation. Knockout of NHE1 reduced cell migration. The effects of a series of novel NHE1/uPA hexamethylene-amiloride-based inhibitors with varying efficacy towards NHE1 and uPA were examined on prostate cancer cells. Inhibition of NHE1—alone, or with inhibitors combining NHE1 or uPA inhibition—generally did not prevent prostate cancer cell migration. However, uPA inhibition—but not NHE1 inhibition—prevented anchorage-dependent colony formation. Application of inhibitors at concentrations that only saturate uPA inhibition decreased tumor invasion in vivo. The results suggest that while knockout of NHE1 affects cell migration, these effects are not due to NHE1-dependent proton translocation. Additionally, while neither NHE1 nor uPA activity was critical in cell migration, only uPA activity appeared to be critical in anchorage-dependent colony formation of DU 145 prostate cancer cells and invasion in vivo.

## 1. Introduction

Prostate cancer is the most commonly diagnosed cancer and second leading cause of death from cancer in men over the age of 60 [1]. Like many solid tumors, prostate cancer can be effectively treated when the disease is confined to the primary organ. However, when the cancer becomes an invasive, metastatic carcinoma, it is often fatal. Patients with prostate cancer are mostly killed by the tumor, which metastasizes to critical organs such as the lungs, liver, or bones; they are not killed directly by the cancer in the prostate [2]. For this reason, it is of utmost importance to identify mechanisms that contribute to the process of cell migration and invasion, and to identify possible targets for therapy [3]. A number of factors contribute to the invasive properties of cells; one of these is the tumor microenvironment. Tumor microenvironments differ from other cell microenvironments, and are often hypoxic or anoxic. They have reduced glucose and ATP levels, along with raised extracellular lactate levels and greater acidity in their extracellular pH (pHe) [4,5,6]. The elevated, alkaline intracellular pH (pHi) is required for growth, proliferation, and motility [7,8], and cancer cells have strategies that work to alleviate intracellular acidification, raise intracellular pH, and acidify extracellular pH. Extracellular matrix degradation is critical for tumor cell invasion; it requires protease-dependent proteolysis at invadopodia sites, where degradation of the extracellular matrix occurs [9].

The pH-related abnormalities are a specific hallmark of malignancy [10]. Dysregulation of pH occurs, creating a reversed pH gradient with constitutively increased intracellular pH, which is greatly elevated above the extracellular pH. This facilitates various characteristics of cancers, including cell proliferation, evading apoptosis, and facilitating metabolic adaptation, which are obligatory for cancer cells. Furthermore, cellular migration is enhanced [7]. A number of acid-extruding proteins exist in tumor cells that aid in this abnormal pH gradient; one critical type is the Na^+^/H^+^ exchanger. Na^+^/H^+^ exchangers are suggested to be important in a number of types of cancers. For example, in breast cancer cells, cytoplasmic alkalinization and extracellular acidification occur due to increased NHE1 (Na^+^/H^+^ exchanger isoform 1) activity [11,12]. Knockout of the NHE1 protein in triple-negative breast cancer cells, such as MDA-MB-231, impairs migration and invasive behavior [13]. NHE1 has also been shown to be important in fibroblasts [14] and the primary cilium [15].

NHE1 has been examined to only a limited degree in prostate cancer. NHE1 mRNA is elevated in prostate cancer [16]. Expression of NHE1 in DU 145 prostate cancer cells correlates with Zeb1 expression. Zeb1 is a transcription factor that promotes epithelial–mesenchymal transition (EMT) which is important in the progression and metastasis of prostate cancer [3,17,18,19]. Hepatocyte growth factor is found in the prostate tumor microenvironment; it triggers invasion, metastasis, and EMT, and also induces NHE activity in DU 145 prostate cancer cells [20].

Another important regulator of prostate cancer cell movement is the urokinase plasminogen activator (uPA) receptor—a trypsin-like serine protease responsible for the activation of plasminogen at the pericellular space [21]. The plasminogen activation system is comprised of uPA, its cell surface receptor uPAR, and two endogenous serpin inhibitors—plasminogen activator inhibitors 1 and 2. Several studies have shown that uPA expression is elevated in patients with prostate cancer [22,23,24].

In this study, we examined the effects of the inhibition of NHE1 and uPA on prostate cancer cell migration and colony formation. Amiloride, the K^+^-sparing diuretic, has previously been shown to inhibit DU 145 and LNCaP tumor growth in xenografted SCID mice when given at high doses [25]. Amiloride is a low μM inhibitor of both uPA and NHE1; however, efforts to repurpose it as an anticancer therapeutic have been thwarted by its antikaliuretic potency and consequent low maximum daily doses [26]. We tested a set of newly described 6-substituted hexamethylene amiloride derivatives with varying potency on one or both of uPA and NHE1. Crucially, these derivatives lack the K^+^-sparing effects of amiloride in vivo, making them better candidates for the development of new cancer drugs for these targets [27]. Here, we examine their effects on the NHE1 activity, cell migration, and colony formation of prostate cancer cells, and additionally examine the roles of NHE1 and uPA in these cells. The results demonstrate that while the presence of NHE1 itself is important in cell migration, its activity is not required. Additionally, while uPA activity is not essential for cell migration, it significantly influences colony formation and invasion in vivo.

## 2. Results

**Figure 1:**
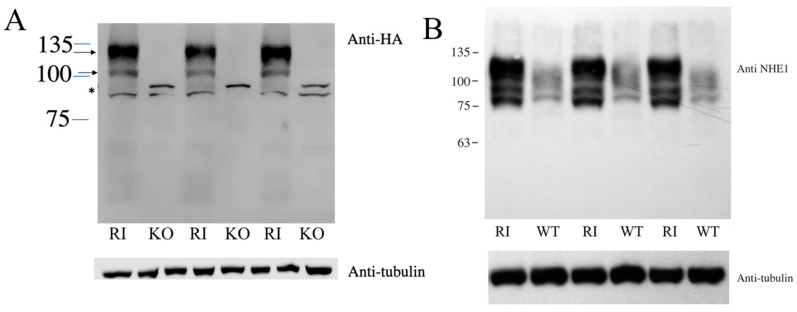
Western blot of DU 145 cell extracts with anti-NHE1 or anti-HA tag antibody. (**A**) Western blot of DU 145 cell extracts probed with anti-HA tag antibody. KO: DU 145 cells with NHE1 knocked out; RI: KO cells with HA-tagged NHE1 returned. Arrows indicate NHE1, * indicates non-specific band. Lower panel probing with anti-tubulin antibodies. (**B**), Western blot of DU 145 cell extracts probed with anti-NHE antibody. WT: wild-type DU 145 cells; RI: KO cells with HA-tagged NHE1 returned. Probed with anti-NHE1 antibodies. Lower panel probing with anti-tubulin antibodies.

Both NHE1 and uPA are known to promote metastasis in various cell types. We therefore examined their role in two cell lines—DU 145 and HT1080 fibrosarcoma cancer cell lines. To examine the role of NHE1 in the migration of DU 145 cells, we deleted the protein using the CRISPR/Cas9 system. To confirm the knockout of NHE1, we used Western blot analysis. Supplementary Figure S1 shows a Western blot of WT and knockout cells using anti-NHE1 antibody. The wild-type DU 145 cells showed two major diffuse bands of ~100 kDa and slightly larger in size, consistent with our observations shown earlier. The larger band corresponds to the full-length glycosylated protein, while the smaller band corresponds to the partial or deglycosylated NHE1 protein [28]. A smaller nonspecific immunoreactive band was present both in the wild-type and knockout cells, as well as in other cell types [29], and for unknown reasons was more abundant in the knockout cells. Re-probing the blot with anti-tubulin antibodies confirmed that the cell extract from knockout cells was present in equivalent amounts. Figure 1A shows the results of a Western blot of KO cells and RI cells with NHE1 reintroduced into the KO cell line and probed with anti-HA tag antibodies. RI cells displayed the typical immunoreactivity of two bands with the anti-HA tag antibody, which was not apparent in the KO cell line. KO cells displayed only 1-2 nonspecific bands of approximately 90–85 kDa (Figure 1A). To compare the relative levels of NHE1 in the WT and RI cells, we probed a Western blot (Figure 1B) with cell extracts from each of these cell types with anti-NHE1 antibody (as opposed to the anti-HA tag antibody in Figure 1A). The RI cells showed very strong immunoreactivity of an upper band, along with some smaller products. The wild-type cells showed much less signal, though there was a definite reproducible signal. The largest immunoreactive band was slightly smaller than in the RI cells, likely due to the addition of the HA tag on NHE1 in the RI cells.

**Figure 2:**
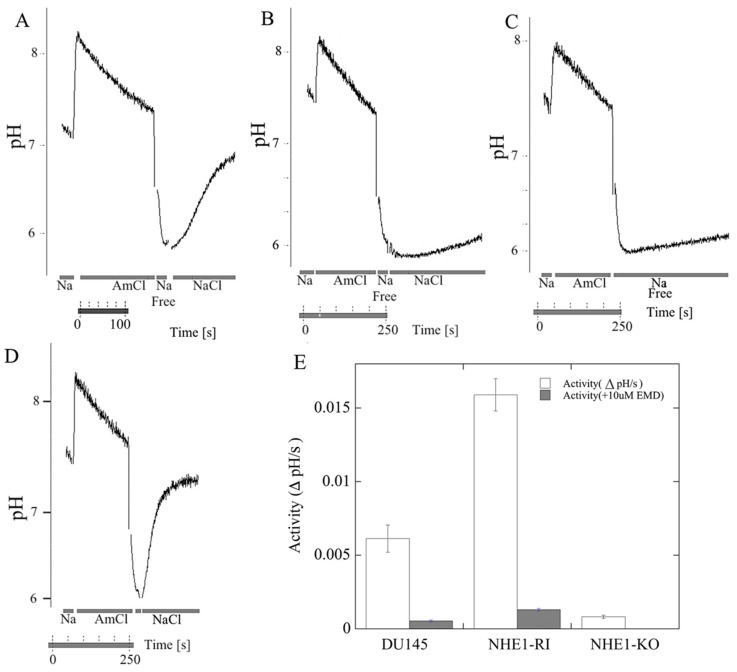
Na^+^/H^+^ exchanger activity in parental DU 145 cells, in NHE1-knockout cells (NHE1-KO), and in knockout cells that had NHE1 reintroduced (NHE1-RI). (**A**–**D**) Examples of traces of activity; these illustrate recovery from an acutely induced acid load. The intracellular pH was recorded in cells transiently acidified using ammonium chloride addition. Periods when NH_4_Cl, NaCl, and Na-free solution were added are indicated. (**A**) wild-type DU 145cells; (**B**) KO cells; (**C**) wild-type cells recovered from acid load in the absence of Na^+^; (**D**) RI: KO cells with NHE1 reintroduced (knock-in). (**E**) Bar graph showing a summary of the initial rate of recovery; this was measured relative to the wild-type protein and recorded as delta pH/s. *n* ≥ 6, mean +/− SE. A pH-sensitive indicator dye, BCECF, was used to measure changes in pH_i_ after the acute acid load induced by the addition and withdrawal of ammonium chloride. The activity of NHE1 was calculated from the changes over the first 20 s of recovery from the acid load, and was expressed as ΔpH/s. Where indicated in (**E**), 10 µM EMD87580 (EMD) was included in the recoveries to confirm that activity was from Na^+^/H^+^ exchanger activity.

In the next series of experiments, we used NHE1 activity assays to examine the activity of wild-type DU 145 cells and the knockout cell line. Figure 2A shows an example of such an assay for NHE1 activity. For the wild type, it illustrates the initial pH, alkalinization caused by ammonium chloride, and the recovery from acidification caused by the removal of ammonium chloride [30]. The recovery upon reintroduction of NaCl is illustrated for both wild-type and knockout cells (Figure 2A,B), and a summary of six experiments is shown in Figure 2E. For wild-type cells, the recovery was robust, at a rate of over 0.008 pH units/s. For knockout cells, the recovery was small and significantly reduced to a level that was approximately the same as the rate of recovery without the addition of NaCl (Figure 2C), as we have observed previously [13,30]. This may be due to either leakage out of the cell [13], or to the action of other proton transporters present in cells [31]. Figure 2D illustrates an example of the recovery of cells with NHE1 returned to the KO cell lines (RI: reintroduced cells). Figure 2E shows a summary of a series of experiments on the initial rate of recovery of the cells lines with and without NHE1. DU 145 cells show robust NHE1 activity, which is eliminated by the NHE1 inhibitor EMD87580 (10 µM). With the reintroduction of NHE1 into the cells, the reintroduced cell line (RI) had even more robust activity, which was eliminated by 10 µM EMD87580. Therefore, EMD87580 (also known as rimeporide) completely inhibited the activity of NHE1 in both the DU 145 wild-type cells and the RI cells. The KO cell line showed virtually no Na^+^/H^+^ exchanger activity.

**Figure 3:**
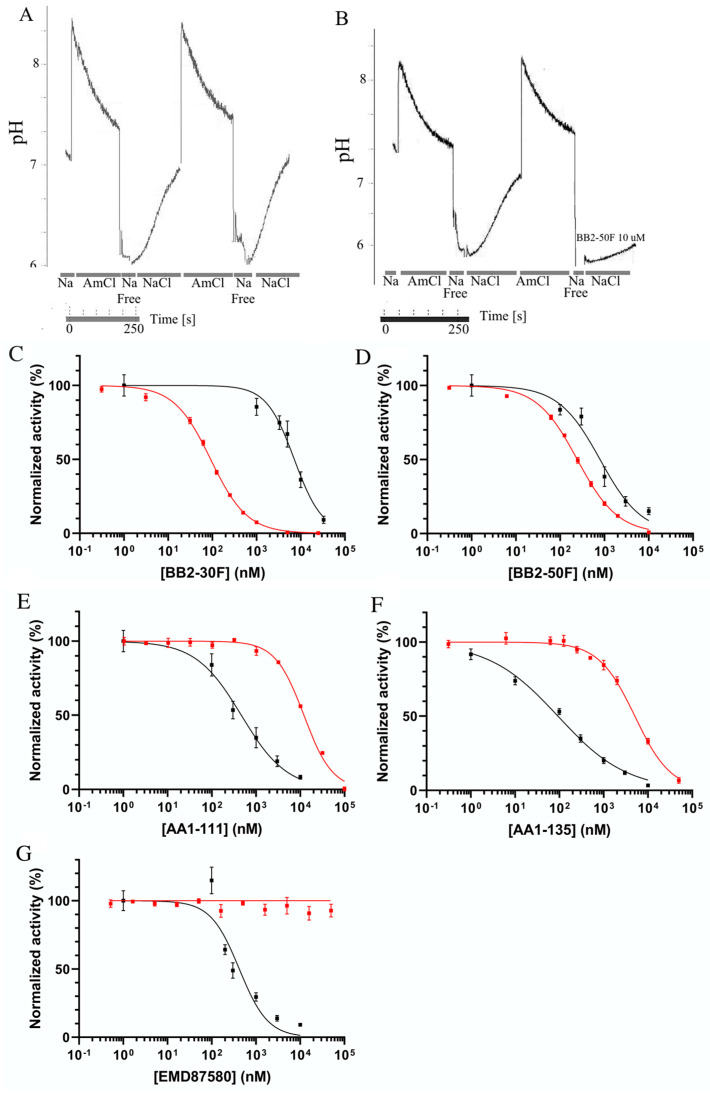
Characterization of the effects of pharmacological compounds on the activity of the Na^+^/H^+^ exchanger using the double-pulse assay. Na^+^/H^+^ exchanger activity was determined as described in Figure 2 and in the Section 4, using a dual ammonium chloride pulse assay. The rate of recovery in the first pulse, in the absence of the drug, was measured. This was compared to the rate of recovery in the second pulse, in the presence of the drug. When both pulses were in the absence of the drugs, the two recoveries were equivalent. (**A**) A double-pulse assay; the second pulse was treated with vehicle only (no drug). (**B**) A double-pulse assay wherein the second pulse was in the presence of 10 µM BB2-50F. (**C**–**G**) A summary of the results of the double-pulse Na^+^/H^+^ exchanger assays (black line) for the compounds AA1-135, AA1-111, BB2-30F, BB2-50F, and EMD87580, respectively (IC_50_ values in Table 1). Additionally, the same figures (**C**–**G**) show the effects of these compounds on uPA activity (red line), assayed as describe d in the Section 4.

We next tested the efficacy of a new family of NHE inhibitors on the activity of the NHE1 protein in DU 145 cells. In order to do this, we used a double-pulse assay, in which there were two consecutive ammonium-chloride-induced acidifications [32]. The rate of recovery in the second pulse in the presence of the inhibitor was measured relative to that of the first pulse in the absence of the inhibitor. In control experiments, we observed that the second pulse, in the absence of the inhibitor, was equivalent to that of the first (Figure 3A). A second example (Figure 3B) shows the effect of 10 µM BB2-50F on the rate of recovery after acute acidosis induced by ammonium chloride. The second rate of recovery, in the presence of the inhibitor, was greatly reduced. A summary of the results is shown in Figure 3C–G, [32] and a summary of the IC_50_ values can be found in Table 1. The various compounds tested had differing potencies towards NHE1 activity. Of the novel compounds, AA-135 was the most potent towards NHE1 (IC_50_ 0.09 µM), while AA1-111 was next (0.45 µM), followed by BB2-50F (0.79 µM), and the least potent was BB2-30F (7.2 µM). EMD87580 was equivalent in potency to AA1-111 (0.45 µM). Figure 3C–G also show the inhibition of uPA activity for each compound on the same plots (red line), assayed as indicated in the Section 4. EMD87580 had no inhibitory activity towards uPA; however, other compounds displayed varying potencies, with AA1-135 and AA1-111 displaying lower potency, and BB2-30F and BB2-50F showing much higher potency, consistent with previous reports [32] (Table 1).

**Figure 4:**
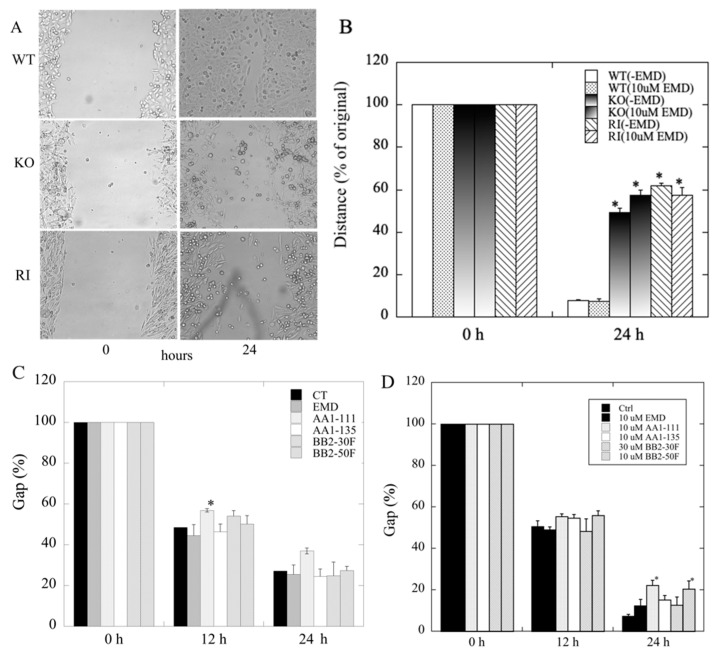
Effects of NHE1-KO or NHE1 inhibition on cell migration examined in wild-type (WT) and NHE1-knockout (KO) DU 145 cells. The rate of closure of the induced gap was evaluated using the wound-healing assay, as described in the Section 4. (**A**) Examples of the effects of NHE1-KO on wound closure. Upper panel: example of WT DU 145 cells with gaps at 0 and 24 h. Middle panel: example of DU 145 NHE1 KO cells at 0 and 24 h. Lower panel: example of RI cell growth. (**B**) Summary of results; * indicates significant differences from the same group at 0 h, *p* < 0.05, *n* ≥ 6. (**C**,**D**) Effects of different concentrations of various NHE1/uPA inhibitors on migration (gap closure) of DU 145 cells. CT: control; EMD: EM87580; other compounds are as names. (**C**) Summary of the effects of 2 µM of various compounds on the migration of wild-type DU 145 cells. (**D**) Summary of the effects of 10 µM of various compounds on the migration of wild-type DU 145 cells (BB2-30F was used at 30 µM); * indicates significant differences from untreated control cells at *p* < 0.05.

To ascertain the role of NHE1 in cell migration, we next examined the effect of knockout of NHE1 on migration of DU 145 cells. The results (Figure 4A,B) demonstrated that knockout of NHE1 significantly reduced the ability of DU 145 cells to close the artificially made gap in wound-healing assays. There were also two unexpected results: The inhibitor of NHE1 activity, EMD87580, did not reduce migration in the any of the groups, including the wild-type and the RI cells. Additionally, the RI cells had reduced migration compared to the wild type (Figure 4B), and equivalent to the KO cells. To confirm this observation, we compared the migration of four different, independently made RI cell lines to that of wild-type cells (Supplementary Figure S2). All of the independently made RI cell lines had their migration ability reduced compared to the wild-type cells. The fourth RI cell line showed less elevated NHE1 activity than the other three cell lines (not shown), and had a more intermediate migration rate between that of the wild-type and the other RI cell lines.

The family of newly developed inhibitors had specific and varying efficacies towards NHE1 and uPA. To determine the effect of NHE1 inhibition in combination with uPA inhibition, we assayed the effects of different concentrations of a number of these compounds on the migration of DU 145 cells. Figure 4C shows the effects of 2 µM EMD87580, AA1-111, AA1-135, BB2-30F, and BB2-50 F; Supplementary Figure S3 shows some examples. Only 2 µM AA1-111 significantly slowed cell migration after 12 h; this effect was relatively small. There was a slight slowing of the migration of AA1-111 at 24 h, although this was not statistically significant, as it was more variable than that at 12 h. Figure 4D shows the effects of 10 µM of these compounds (and 30 µM BB2-30F) at 12 and 24 h. At 12 h there were slight but non-significant effects of AA1-111, AA1-135. and BB2-50F; this became significant only for AA1-111 and BB2-50F at 24 h, but the effect was not large. The other compounds—EMD87580, AA1-135, and BB2-30F—had only very slight, non-significant effects on gap closure.

**Figure 5:**
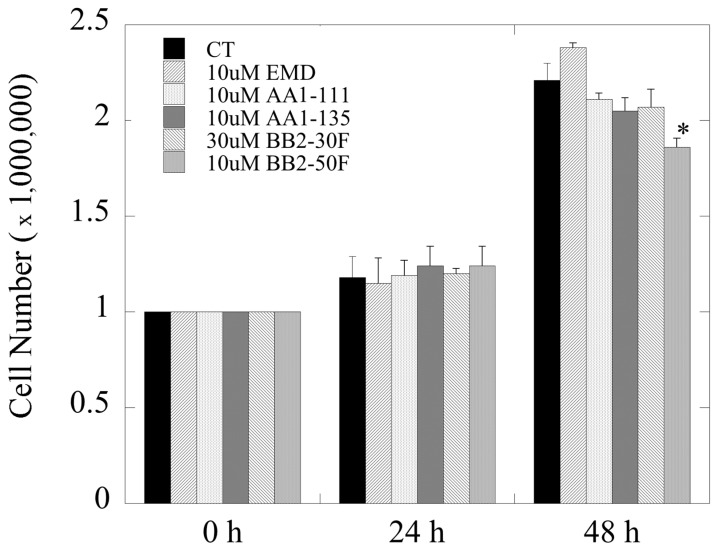
Effects of pharmacological compounds on the growth of DU 145 cells. Cell growth was quantified as described in the Section 4; * indicates significant differences from untreated control cells at *p* < 0.05.

We examined the effects of the higher concentrations of these compounds (used in Figure 4D) on the growth of DU 145 cells. Over 24 h there was no significant effect on growth with any of the compounds. Over a period of 48 h (Figure 5), there was only one slight significant effect of 10 µM BB2-50F on cell growth. 

**Figure 6:**
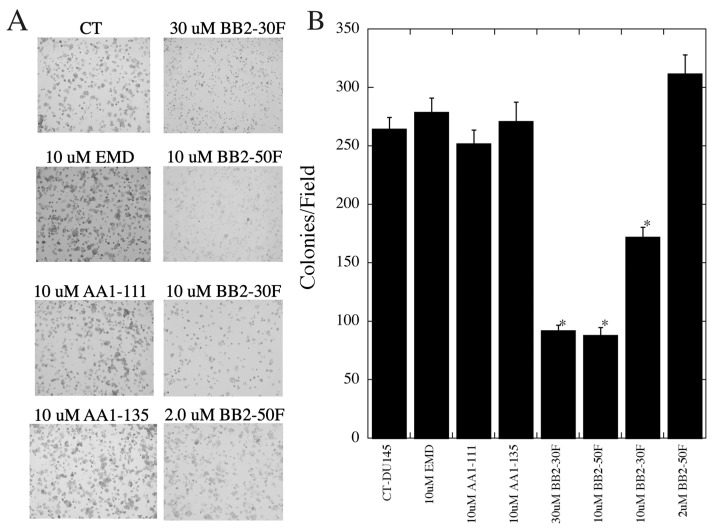
Effects of pharmacological compounds on anchorage-dependent colony formation of DU 145 cells. (**A**) Examples of results. (**B**) Summary of mean +/− SE of 4 experiments. Colony formation was measured as described in the Section 4; * indicates significant differences from untreated control cells at *p* < 0.05.

We then examined the ability of these compounds to inhibit anchorage-dependent growth of the DU 145 cells (Figure 6A,B). EMD87580, AA1-111, and AA1-135 at a concentration of 10 µM had no effect on colony formation. BB2-30F inhibited colony formation at concentrations of both 10 and 30 µM, though the effect was more pronounced at 30 µM. In both cases, colonies formed, but were very small and below measurement criteria for inclusion as a colony. BB2-50F was used at 2 and 10 µM, and was inhibitory to colony formation at 10 µM, but not at 2 µM; at the higher concentration there were also small colonies present.

**Figure 7:**
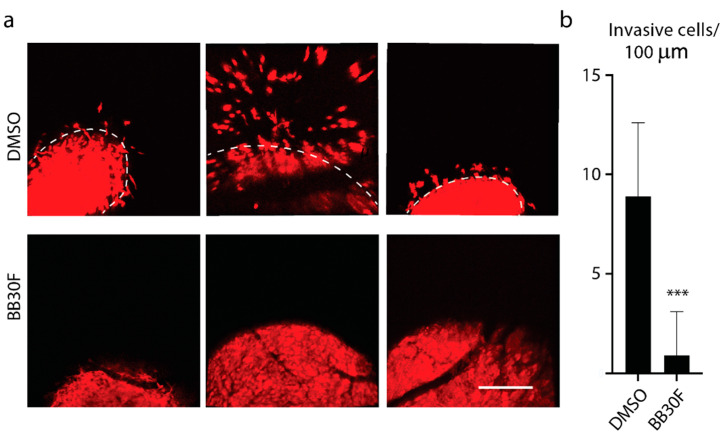
Intravascular intravenous application of BB2-30F compound blocks to HT1080 human fibrosarcoma cancer cell invasion in vivo. (**a**) Representative images of invasive tumor fronts of DMSO-treated controls (upper row) and BB2-30F-treated HT1080 tumor fronts. (**b**) Quantification of invasive tumor cell numbers for conditions in (**a**). Dashed lines in (**b**) delineate the primary tumor borders. Scale bar = 100 µm; *** indicates significant difference at *p* < 0.0001.

Our next set of experiments used a different cell line and an in vivo model. We examined whether BB2-30F was capable of blocking tumor cell invasion from primary tumors in vivo, utilizing our previously established chick chorioallantoic membrane model of human cancer progression [33,34]. HT1080 cells were allowed to grow tumors for 4 days, and the drug treatment with BB2-30F was started 1 day after tumor cell inoculation. Control, DMSO-treated tumors displayed robust cancer cell invasion at the primary tumor fronts, with multiple tumor cells invading into the chorioallantoic membrane tissue (Figure 7a,b). In contrast, treatment with a final concentration of 0.5 µM BB2-30F produced near-complete inhibition of tumor cell invasion (Figure 7a,b).

## 3. Discussion

Prostate cancer represents over 7% of all malignancies diagnosed in men. The majority of deaths occur due to metastasis, which occurs when castration-resistant prostate cancer spreads. Therapies are underway to improve androgen deprivation treatment, including treatment with androgen-receptor-axis-targeting agents such as apalutamide, abiraterone, and enzalutamide. Though these agents show significant efficacy, there is clearly room for more treatments in this area [35], as well as for the development of treatments to prevent metastasis.

In this contribution, we studied putative regulators of cell migration and invasion—NHE1 and uPA—in the DU 145 prostate cancer cell line. Additionally, we tested the effects of a series of novel amiloride derivatives with either single or dual targeting for the inhibition of isoform one of the Na^+^/H^+^ exchanger and uPA [32]. We also examined the effects of uPA inhibition in vivo on the HT1080 fibrosarcoma cancer cell line. The DU 145 cell line is one of the most commonly used human prostate cancer cell lines in therapeutic research, and is androgen-receptor-containing, with moderate metastatic potential [36]. A number of earlier studies have suggested that the tumor microenvironment, proton flux, and uPA [31] may be important in prostate cancer, as well as in tumorigenicity in prostate cancer and in the aforementioned cell lines [36]. In the case of the tumor microenvironment, a more alkaline intracellular pH (pHi) is known to be required for growth, proliferation, and motility, and this is accompanied by a decreased extracellular pH [7,8]. For prostate cancer cells, recent reviews summarize the evidence that proton flux from within the cell to the cell exterior alkalinizes the cell interior and acidifies the cell exterior, both of which contribute to the migration, invasion, and metastasis of prostate cancer cells [31,37,38].

NHE1 was chosen for investigation here for several reasons. Expression of NHE1 in DU 145 prostate cancer cells correlates with Zeb1 expression [3,17,18,19]. Zeb1 is an important transcription factor that promotes epithelial–mesenchymal transition (EMT), which is important in the progression and metastasis of prostate cancer [3,17,18,19]. Hepatocyte growth factor is found in the prostate tumor microenvironment; it triggers invasion, metastasis, and EMT, and induces NHE activity in DU 145 prostate cancer cells [20]. Multiple NHE isoforms have been shown to be involved in prostate cancer cells’ extracellular lysosome trafficking and extracellular acidification [39]. In other types of cancer, such as breast cancer, deletion of NHE1 inhibits invasion in vivo and in vitro, and elevated levels and activity of NHE1 promote metastasis and invasion [40]. Thus, we chose NHE1 and uPA as dual targets in this investigation of prostate cancer cells.

The results were interesting in both cases, though not entirely as expected. We were able to successfully knock out the NHE1 isoform of the Na^+^/H^+^ exchanger in this cell line, and the cells were viable. Knockout of the NHE1 protein had a large effect on cellular migration, as demonstrated in our wound-healing assays. However, there were some surprising features of this observation. The effect on migration did not appear to be due to the lack of NHE1 activity. Two lines of evidence support this conclusion: Firstly, inhibition of NHE1 by EMD87580 (Figure 4B) or by other inhibitors (Figure 4C) at concentrations well above their IC_50_ for NHE1 failed to have significant effects on cell migration. The only minor exception to this observation was the effect of AA1-111 (Figure 4C), which was small in comparison to the effect of KO of NHE1.

The second piece of evidence that NHE1 activity per se was not critical to migration was that when we returned NHE1 to the KO cells, the migration was not restored to normal (Figure 4B). This was an unusual result, especially since we had confirmed both the return of the NHE1 protein (Figure 1) and its activity, which was inhibited by the NHE1 inhibitor EMD87580 (Figure 2). The activity of the RI cells was greater than that of wild-type DU 145 cells (Figure 2E). It should be noted that the inhibition of NHE1 activity in the RI DU 145 cells with EMD87580 did not improve migration (Figure 4B), so this effect was not due to excessive protein activity. Interestingly, these data contrast with earlier work in breast cancer cells, where knockout of NHE1 inhibited the migration of cells, but its reintroduction restored cell migration and invasion. Additionally, the return of more active NHE1 enhanced cell migration and invasion [40]. Clearly, the role that NHE1 plays in these two cancer cell types varies, being more important in breast cancer cells in comparison with prostate cancer cells. It may be the case that in prostate cancer cells, the activity of another Na^+^/H^+^ exchanger plays a more critical role than NHE1. Steffan et al. [20,39] have shown that the NHE3 isoform plays an important role in acidifying the extracellular pH of prostate cancer cells, and promotes lysosomal trafficking and exocytosis. It is also of interest that NHE1 knockout inhibited cell migration, but its inhibition did not. NHE1 has more roles than just regulation of intracellular pH; it acts as a scaffold for other proteins and a link to the cytoskeleton [14]. We suggest that some of NHE1′s other functional roles, aside from pH regulation, are critical in migration—possibly its linkage to the cytoskeleton, which has been implicated in this regard in some cell types [14].

Exogenous NHE1 was expressed at a higher level than the endogenous protein (Figure 1B). An explanation of why this was inhibitory to cell migration is not yet clear, but there are several possibilities. NHE1 acts as a scaffold; it is both a substrate for protein kinases and also binds protein kinases [41,42]. Mislocalized scaffolding by NHE1 has earlier been shown to affect intracellular signaling [43]. It is possible that overexpression of the NHE1 protein caused aberrant intracellular signaling. It is noteworthy that overexpression of NHE1 has been shown to activate the Rho kinase Rock, and has detrimental effects in induced pluripotent stem cells [44]. NHE1 is also a scaffold; it binds ERM (ezrin, radixin, moesin) proteins [45] and other intracellular proteins, such as calmodulin [46], calcineurin homologous protein [47], and carbonic anhydrase [48]. These proteins do not exclusively interact with NHE1, and overexpression of NHE1 may affect other physiological functions that affect cell migration. ERM proteins influence plasma membrane protrusion, cell substrate adhesion, and cortical actin organization [49,50], and NHE1 overexpression can result in disorganized cyst formation of MDCK cells [51], and can mediate morphological organizational changes that occur in melanoma cells [52]. It seems that in DU 145 cells, the precise level of NHE1 is critical. Deletion of the protein has detrimental effects on cell migration, but overexpression also has detrimental effects. The presence of NHE1 appears to have a biphasic effect in prostate cancer cells; though its activity is not required, its presence appears to play another role, as demonstrated by its removal, which inhibits cell migration. However, when reintroduced at higher levels, it is also inhibitory. This is in contrast to breast cancer cells [40], in which overexpression and hyperactivity of NHE1 promote metastasis and invasion. Here, we show that this is not the case in prostate cancer cells.

We also examined the effects on the migration of DU 145 cells of two different concentrations of a series of inhibitors that were based on the amiloride analog hexamethylene amiloride [27,53]. Two different doses of compounds were used. A lower concentration of 2 µM was chosen. For AA1-111, AA1-135, and EMD87580, this concentration provided complete NHE1 inhibition with minimal inhibition of uPA (Table 1). For BB2-50F and BB2-30F, 2 µM provided good inhibition of uPA and only partial inhibition of NHE1 for BB2-50, and low inhibition of NHE1 for BB2-30F. The only compound that provided a significant effect at this concentration was AA1-111, which reduced cell migration slightly. While theoretically this could be NHE1-dependent, the lack of effect of AA1-135 and EMD87580 suggests that the effect is not likely to be due to inhibition of NHE1 activity.

The higher concentrations of compounds—10 µM for all except BB2-30F (30 µM)—were chosen to have effects on the complementary activities. For BB2-30F and BB2-50F, the higher concentrations affected NHE1. For AA1-111 and AA1-135, there were be more effects on uPA. EMD87580 had no effect on uPA (Figure 3G), even at high concentrations. With all of these compounds, there were no significant effects on cell migration. AA1-111 again had an effect comparable to that shown at 2 µM concentrations (Figure 4C), though this was slightly more variable in these experiments and was not statistically significant. Overall, these results suggest that—at least for this set of compounds, and in a cell migration assay—inhibition of NHE1 or uPA is not effective at preventing cell migration, and dual inhibition of NHE1 and uPA did not improve any inhibitor effects on cell migration.

The effects on colony formation were more interesting than the effects on cell migration. The NHE1 inhibitor EMD87580, which does not inhibit uPA, had no significant effect on anchorage-dependent colony formation. Similarly, the same concentrations of AA1-111 and AA1-135 did not affect colony formation (Figure 6), and at these concentrations these compounds completely inhibited NHE1, with only minimal inhibition of uPA activity. Clearly, NHE1 activity is not critical for anchorage-dependent colony formation. Interestingly, both BB2-30F and BB2-50F were able to inhibit colony formation. For BB2-30F, both 10 and 30 µM significantly reduced colony formation. In both cases, uPA inhibition was typically complete, and NHE1 inhibition only partial—especially at the 10 µM concentration. BB2-50F only inhibited colony formation at a concentration of 10 µM, and not at 2 µM. However, the IC_50_ of BB2-50F for uPA is more than double that of BB2-30F, which may account for the incomplete uPA inhibition at the 2 µM concentration. It should be noted that BB2-50F did inhibit cell growth at the 10 µM concentration with treatment for 48 h, though this effect was relatively small. This result is consistent with previous findings where BB2-50F showed half-maximal inhibition of cell viability in the 4-13 µM range in a panel of established transformed human cell lines [27]. Overall, these experiments suggest that uPA activity plays a significant role in anchorage-dependent colony formation, while NHE1 activity is not required. This conclusion was supported by in vivo experiments in chick embryos. Intravenous application of BB2-30F blocked HT1080 human fibrosarcoma cancer cell invasion in vivo. A relatively low final concentration of uPA in the embryo was used—0.5 µM, which is well below the IC_50_ for NHE1 of 7.2 µM (Table 1). uPA is important in several types of cancer, including lung and pancreatic cancer metastasis [27], and knockdown of uPA has been shown to inhibit metastasis of MDA-MB-231 breast cancer cells [54]. The uPA receptor is important in the growth and metastasis of nasopharyngeal carcinoma cells [55], and uPA enhances cell invasion and adhesion of ovarian cancer cells [56]. In prostate cancer, uPA is suggested to be important in the establishment of prostatic epithelial cells in bone marrow [57]. As noted above, several studies have shown that uPA expression is elevated in patients with prostate cancer [22,23,24]. Our study supports a role for uPA in prostate cancer cell metastasis, and is supportive of proposed targeting of uPA in prostate cancer [58]. The mechanism of action of uPA in the regulation of prostate cancer cell movement has yet to be elucidated. However, uPA is known to activate plasminogen to the broad-spectrum plasmin. This acts to degrade the extracellular matrix, and activates a number of downstream regulators such as pro-matrix metalloproteinases, along with the release of growth factors from the extracellular matrix [27]. This might also be the mechanism at play in the cell type under study.

In summary, our study examined NHE1 and uPA in prostate cancer cells. We found that NHE1 appears to be important in cell migration, as suggested by its deletion. However, overexpression of NHE1 was also inhibitory to cell migration. Puzzlingly, inhibition of NHE1 with several inhibitors did not prevent the migration of prostate cancer cells, and it appears that some other physiological roles of NHE1 are important in this cell type, rather than NHE1 activity itself. We examined several NHE1/uPA inhibitors with varying potencies in the inhibition of NHE1 and uPA. While they were not effective in preventing cell migration, colony formation was greatly reduced by inhibitors with potent uPA-inhibitory capacity. In our study, we concentrated on DU145 cells, which are a commonly used cell line in prostate cancer studies. We also verified many of the results in vivo, using the HT1080 fibrosarcoma cell line. DU145 cells are one of the standard cell lines used in therapeutic research, but are not hormone sensitive. Future experiments could examine how widespread the effects are in other prostate cancer cells and other cell types. Further experiments could also explore the roles of these compounds in the prevention of prostate cancer metastasis. It is important to note that our in vitro findings were demonstrated in only one prostate cancer cell line (DU145), and that in vivo verification of these results was performed in a fibrosarcoma model of metastasis. Thus, further studies are needed in order to firmly conclude that these observations are broadly applicable to prostate cancer, or to other cancer types in general.

## 4. Materials and Methods

### 4.1. Materials

Synthetic oligonucleotides were obtained from IDT (Coralville, IA, USA). Sulfo-NHS-SS-biotin was bought from Thermo Fisher (Waltham, MA, USA). 2′,7-Bis(2-carboxyethyl)-5(6) carboxyfluorescein acetoxymethyl ester (BCECF-AM) was obtained from Molecular Probes, Inc. (Eugene, OR, USA). Lipofectamine^TM^ 2000 reagent was obtained from Invitrogen Life Technologies (Carlsbad, CA, USA). Sigma-Aldrich (St. Louis, MO, USA) or Fisher Scientific (Toronto, ON, Canada) supplied the other analytical-grade chemicals used in this study. The plasmid pYN4+ was used to express cDNA for the full-length human NHE1 protein; it has a triple HA (hemagglutinin) tag, is completely functional, and has been described previously [59]. Anti-NHE1 protein antibody (#611774) was purchased from BD Biosciences. Anti-tubulin protein antibody was purchased from Sigma (St. Louis, MO, USA). The plasmid pSpCas9(BB)-2APuro (pX459) V 2.0 was a generous gift from Dr. M. Michalak, Department of Biochemistry, University of Alberta. DU 145 cells and HT1080 cells were obtained from ATCC—catalog numbers HTB-81 and CCL-121, respectively.

### 4.2. Knockout (KO)/Reintroduction (RI) of NHE1

To knock out the human Na^+^/H^+^ exchangers’ isoform 1, we used the CRIPR/Cas9 system. Three sets of primers were chosen and designed with the assistance of the https://zlab.bio/guide-design-resources website (accessed on 1 June 2019) in order to minimize off-target interactions (Table 1).

The three pairs of 100 µM primers (Table 2) were individually annealed at 95 °C for 5 min and then cooled down at room temperature for 6 h. The plasmid pX459 was digested with BbsI for 3 h at 37 °C. Then, the annealed primers and digested pX495 were ligated with T4 DNA ligase overnight at 15 °C. The ligation solution was treated with plasmid-safe DNase for 40 min at 37 °C, and 2 µL of the treated solution was used to transform the Stbl3-competent *E. coli* for screening. Plasmids with appropriate restriction enzyme maps were confirmed by DNA sequencing.

Wild-type DU 145 cells were cultured in incubators at 37 °C with a humidified atmosphere containing 5% CO_2_ in RIPM growth medium supplemented with 10% (*v*/*v*) bovine growth serum, 25 mM HEPES, 100 U/mL penicillin–streptomycin (100 μg/mL), and 50 mg/mL gentamicin at pH 7.4. One day before transfection, 2.0 × 10^5^ cells were grown in 35 mm Petri dishes using 2 mL of growth medium. All three plasmid constructs with the inserted guide sequences (4~6 µg total DNA) were stably transfected into DU 145 cells at 80~90% confluence with Lipofectamine™ 2000 reagent [60]. Puromycin (1 µg/mL) was used to select positive, stably transfected clones. Cells were removed from 35 mm petri dishes with trypsin on the second day after transfection. This was followed by dilution with growth medium to 1:100. Cells were then plated in 100 mm dishes containing 1 µg/mL puromycin in order to maintain selection pressure for 3 days. Single clones surviving from the selection were picked and cultured in 12-well plates. When confluent cells were split, half were put into 6-well plates and half into 35 mm petri dishes. The cells in the 35 mm petri dishes were used to screen for positive clones by immunoblotting. The positive cell lines were grown in 6-well plates and were collected and grown in growth medium. Frozen stocks of cell lines were stored in liquid N_2_. Cell lines were brought up from frozen stocks whenever necessary, between passage numbers 5 and 11. At least two stable cell lines were made independently for each mutant.

To return the human NHE1 protein to the knockout cells, we transfected DU-145-knockout cells with the plasmid pYN4+ that contains human NHE1 cDNA with a HA (hemagglutinin) tag, as described previously [29]. Briefly, cells were transfected with the NHE1-containing plasmid using Lipofectamine™ 2000 reagent, as described above. The plasmid contains a neomycin-resistant cassette, allowing for selection using G418 resistance. Stable cell lines were established as described above, and were re-established from frozen stocks at passage numbers 5-11 when necessary.

### 4.3. SDS-PAGE and Immunoblotting

We examined NHE1 levels in wild-type and knockout DU 145 cell lines via immunoblotting against anti-NHE1 protein antibody where indicated, as described previously [13]. Briefly, SDS-PAGE was used to separate cell lysate proteins in (10%) gels and proteins were transferred to nitrocellulose. Equal amounts of protein from both control and experimental lysates were run in triplicate for examination. The Bio-Rad D/C^TM^ Protein Assay kit was used to measure protein concentrations. The secondary antibody used was peroxidase-conjugated goat anti-mouse antibody (Bio-Can, Mississauga, ON, Canada). To examine the reintroduction of HA-tagged NHE1, we used anti-HA monoclonal antibody [29].

### 4.4. Measurement of Intracellular pH

Intracellular pH was measured using 2′,7-bis(2-carboxyethyl)-5(6) carboxyfluorescein acetoxymethyl ester (BCECF-AM), in a similar manner to that described in [29]. DU 145 cells were grown to ~80–90% confluence on glass coverslips. Cells were loaded with BCECF and fluorescence was measured using a PTI Deltascan spectrofluorometer. NHE1 activity was measured after inducing an acute acid load using ammonium chloride. This was achieved by addition of ammonium chloride (50 mM × 3 min), followed by removal to induce acute acidosis. ΔpH/s was measured during the first 20 s of recovery in NaCl-containing medium (135 mM NaCl, 5 mM KCl, 1 mM MgCl_2_, 1.8 mM CaCl_2_, 5.5 mM glucose, and 10 mM HEPES, pH = 7.4, 37°). A calibration curve of pH_i_ fluorescence was performed for every sample using nigericin [60]. For testing of the effects of inhibitors on NHE activity, a double ammonium chloride pulse protocol was used [61]. Cells were subjected to one cycle of acidification using ammonium chloride, and then were allowed to recover in the absence of a drug, with vehicle where appropriate. Then, another cycle of acidification and recovery followed, with the drug present in both the sodium-free and normal recovery buffers. IC_50_ values were calculated as described previously [61]. The measurements were highly reproducible, with activity in the second pulse averaging 98.2 +/− 7.15% of that in the first pulse. The results are the mean ± S.E. of at least seven experiments. The statistical significance was determined via the Wilcoxon–Mann–Whitney test.

### 4.5. Cell Migration Assay

Cell migration was studied using a wound-healing assay similar to that described in [13]. Briefly, cells were seeded in 12-well plates (4 × 10^6^ cells/well) the day before assay, and then grown to a confluent monolayer. A “scratch” was created in the cell monolayer with a p20 pipette tip in a straight line. The cells were washed twice with PBS to remove the debris, and the medium was changed to 2 mL of growth medium. To obtain the same field during image acquisition, three crosses were marked on the outside of each well as a reference. The scratch gaps above and below the crosses were measured. We acquired six images of the scratch for each well using a Leica DM IRB microscope (at 10× magnification). Arbitrary measurements of gap closure were quantified using Image-Pro Plus software. A minimum of six images/well and five measurements/image were acquired for each treatment; all treatments were repeated at least three times. Data shown are representative of the decrease in gap distance at 24 h, compared to the initial measurement at 0 h. Compounds were added at the indicated concentrations.

### 4.6. Cell Growth Assays

Cell growth was measured in wild-type DU 145, NHE1-KO, and RI cells [13]. Cells were plated into 24-well plates and grown in 10% serum medium overnight before assay, and were approximately 50% confluent. Four wells of growing cells were washed twice with PBS, and then 0.25% trypsin-EDTA was added to detach the cells. After brief centrifugation, the cell pellets were resuspended in 1 mL of medium, and then cells were counted with a microscope using a hemocytometer. The initial cell numbers were obtained during the day. The balance of the cells was detached 24 and 48 h later and counted using the same method. A minimum of four repeats were performed for each experiment; all treatments were replicated at least three times.

### 4.7. Anchorage-Dependent Colony Formation

To examine anchorage-dependent colony formation, we used 60 mm polystyrene dishes that had been coated to introduce hydrophilic groups producing a standard growth surface (Sarstedt, Numbrecht, Germany). To measure anchorage-dependent colony growth, the cells were plated into in the 60 mm dishes at a density of 1000 cells per dish. They were grown in complete growth media. To examine the effects of various compounds, 2–30 µM of the indicated drugs were added to culture dishes. An equal volume of DMSO (vehicle) alone was added to otherwise-untreated controls. After growth for 8 days, colonies were fixed in ice-cold methanol; they were stained with 0.5% crystal violet in methanol and washed, and then left to air-dry. The colonies of > 50 cells/colony were imaged and counted. The number of mutant colonies was normalized to the number of control colonies and presented as a percentage of control colonies formed.

### 4.8. Statistics

All experiments were repeated at least 4 times. Results are presented as the mean +/− SE. Results were plotted with KaleidaGraph 4.1 (Synergy Software, Reading, PA, USA). Statistics for all experiments were measured using a Wilcoxon–Mann–Whitney rank-sum test. A *p*-value less than 0.05 was deemed to be statistically significant. For intracellular pH measurements, the reported results are those of at least 7 experiments per treatment/condition.

### 4.9. Inhibitors

Synthesis of NHE1/uPA inhibitors was as described previously, as was the determination of uPA activity, where each compound was described as the number in parentheses: BB2-50F (5), BB2-30F (26), AA1-111 (29), and AA1-135 (30) [32].

### 4.10. Ex Ovo Chick Embryo Culture

Fertilized White Leghorn chicken eggs were obtained from the University of Alberta’s Poultry Research Centre and maintained in a humidified incubator at 38 °C. After four days of incubation, embryos were removed from their shells using a Dremel rotary tool with a cutting wheel. They were maintained as previously described under shell-less conditions, in a covered dish in a humidified air incubator at 38 °C and 60% humidity [33,34], until 10 days post-fertilization. On the 10th day of development, chicken embryos were inoculated with 1 × 10^5^ HT1080 cells that were genetically engineered to express tdTomato fluorescent protein. For drug treatment, chickens were injected intravenously with DMSO or BB30F to a final concentration of 0.5 µM within the embryo (starting on day 1 after tumor inoculation; 50 µL total injection volume). At least 8 chick embryos were used in each experiment. On day 3 of the drug treatment, sterilized, rounded (22 mm) coverslips were applied on top of the tumor, and invasive tumor fronts were visualized.

### 4.11. Embryo Image Acquisition and Analysis

For imaging, a Nikon A1r upright confocal microscope (Nikon) fitted with a temperature-regulated enclosure and a 10× objective was used (591 nm laser). A 100 µm image stack was acquired using 5 µm step size increments, and the whole z-stack was displayed as maximum intensity projections. These experimental data were plotted and analyzed for statistical significance using the Prizm analysis module.

## Figures and Tables

**Table 1:** Potency (IC_50_ in μM) of various compounds towards NHE1 and uPA.

Compound	BB2-30F	BB2-50F	AA1-111	AA1-135	EMD87580
NHE1	7.2	0.79	0.45	0.09	0.46
uPA	0.09	0.24	12.1	5.1	N/A

**Table 2:** Synthetic oligonucleotides used to knock out the NHE1 protein. Guide sequences are underlined, and added bps used for restriction enzyme cloning are shown in lower case.

Name	Guide Sequence	Target Site
NHE1CRISPR1f	caccgGATCAACAACATCGGCCTCC	Exon 2
NHE1CRISPR1r	aaacGGAGGCCGATGTTGTTGATC	
NHE1CRISPR2f	caccgCGGGACGATGCTTGAGATAG	Exon 2
NHE1CRISPR2r	aaacCTATCTCAAGCATCGTCCCG	
NHE1CRISPR3f	caccgGTTTGCCAACTACGAACACG	Exon 3
NHE1CRISPR3r	aaacCGTGTTCGTAGTTGGCAAAC	

## Data Availability

Not applicable.

## Supplementary Materials

The following are available online at https://www.mdpi.com/article/10.3390/ijms222413263/s1.
