# Supplementary material for: Roles of the Na+/H+ Exchanger Isoform 1 and Urokinase in Prostate Cancer Cell Migration and Invasion
Source: Int J Mol Sci. 2021 Dec 9;22(24):13263. doi: 10.3390/ijms222413263 (PMC8705693; doi:10.3390/ijms222413263)
Supplement: Supplementary file 1 [file ijms-22-13263-s001.zip › ijms-1472990-supplementary.pdf]

International Journal of
Molecular Sciences

Supplementary materials

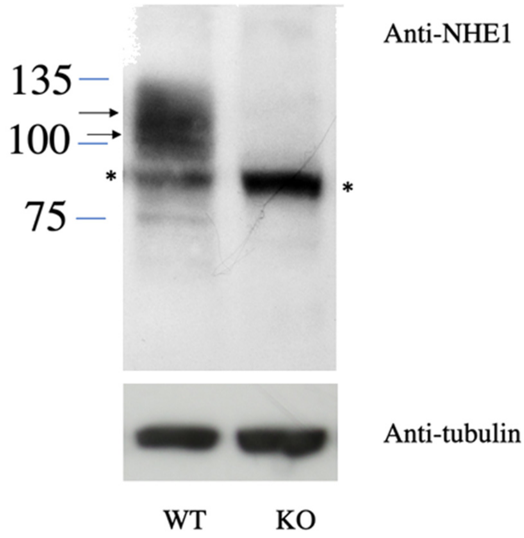

Figure S1. Western blot of DU 145 cell extracts blotted with anti-NHE1. Upper panel, immunoreactivity with anti-NHE1 antibody. Lower panel, immunoblot of same samples with anti-tubulin antibodies. Arrows indicate the positions of the NHE1 protein. Asterisk indicates position of non-specific band. WT, wild type DU 145 cells; KO, DU 145 cells with NHE1 knocked out.

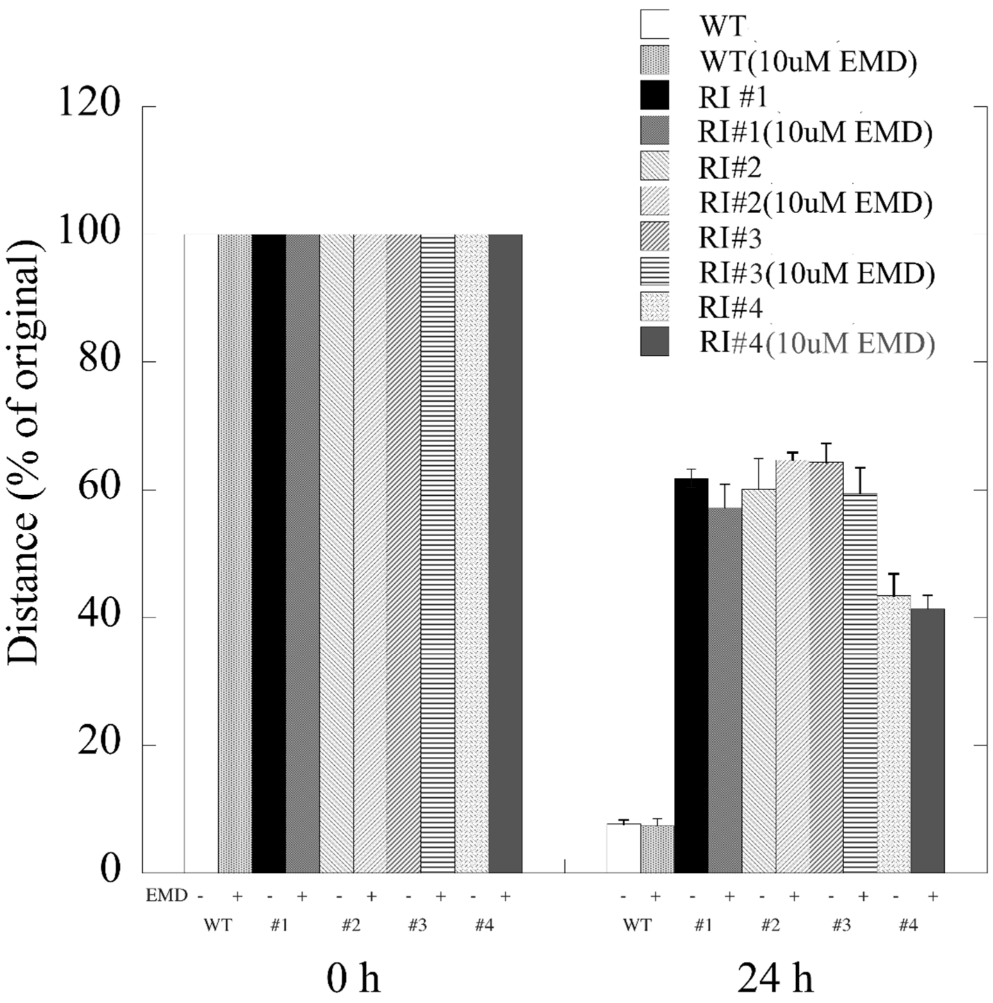

Figure S2. Comparison of rate of migration of 4 different, independently made RI (NHE1 reintroduced) cell lines, to that of wild type cells. The rate of closure of the induced gap was evaluated using the wound-healing assay which is described in Fig. 4. EMD87580 was added at 10 uM final concentration, where indicated.

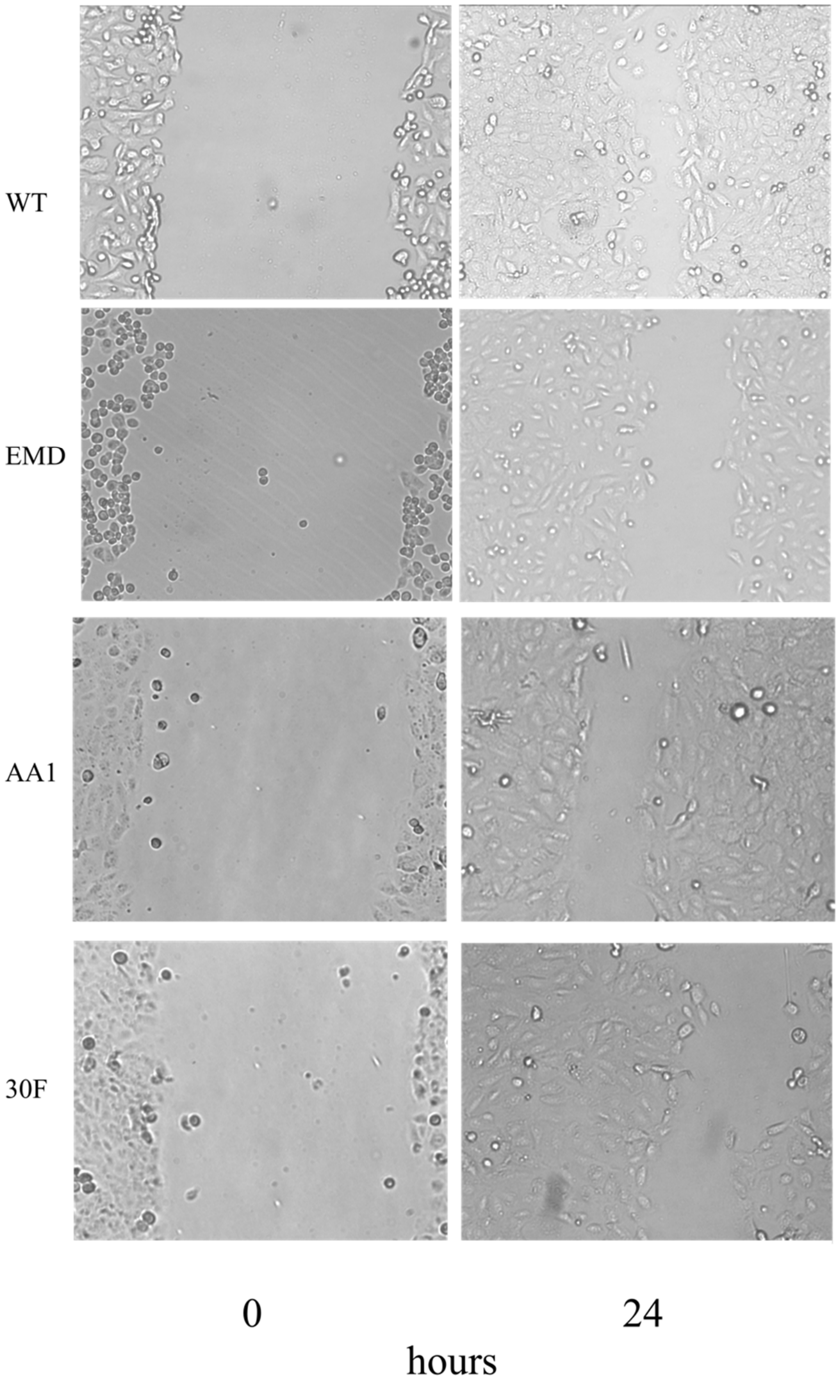

Figure S3. Examples of effect of various inhibitors (10 uM) on cell migration of wild type (WT) DU 145 cells. Gap at 0 and 24 hours is shown. The wound-healing assay is described in the Materials and Methods and Figure 4. EMD, EM87580; AA1, AA1-111; 30F, BB2-30F.
